# Pain in veterans of the Gulf War of 1991: a systematic review

**DOI:** 10.1186/1471-2474-7-74

**Published:** 2006-09-20

**Authors:** Hollie V Thomas, Nicola J Stimpson, Alison Weightman, Frank Dunstan, Glyn Lewis

**Affiliations:** 1 Department of Psychological Medicine, School of Medicine, Cardiff University, UK; 2 Information Services, School of Medicine, Cardiff University, UK; 3 Department of Epidemiology, Statistics and Public Health, School of Medicine, Cardiff University, UK; 4 Academic Unit of Psychiatry, University of Bristol, UK

## Abstract

**Background:**

Veterans of the Persian Gulf War of 1991 have reported a range of adverse health symptoms. This systematic review aims to identify all studies that have compared the prevalence of symptoms of pain in veterans of the Gulf War to that in a non-Gulf military comparison group, and to determine whether Gulf War veterans are at increased risk of reporting pain.

**Methods:**

Studies published between January 1990 and May 2004 were identified by searching a large number of electronic databases. Reference lists and websites were also searched and key researchers were contacted. Studies were included if they reported the prevalence of any symptom or condition that included the word "pain" in Gulf War veterans and in a comparison group of non-Gulf veterans. 2401 abstracts were independently reviewed by two authors.

**Results:**

Twenty studies fulfilled the inclusion criteria. Five main sites of pain were identified (muscle, joint, chest/heart, back and abdominal pain) and separate meta-analyses were performed to summarise the results related to each site. A greater proportion of Gulf veterans reported symptoms at each site of pain when compared to a non-Gulf military group. Gulf deployment was most strongly associated with abdominal pain, with Gulf veterans being more than three times more likely to report such pain than a comparison group (OR 3.23; 95%CI 2.31–4.51). Statistical heterogeneity between study estimates was significant, probably due to variation in measured periods of prevalence and symptom measurement methods.

**Conclusion:**

A higher proportion of veterans of the Persian Gulf War of 1991 reported symptoms of pain than military comparison groups. This is consistent with previously demonstrated increased reporting of more general symptoms (fatigue, multiple chemical sensitivity, post traumatic stress disorder) in these veterans compared with non-Gulf military groups. However, the primary studies were heterogeneous and varied greatly in quality.

## Background

Shortly after returning from the Gulf War in 1991, veterans started to report a range of adverse health symptoms [1]. One of these reported symptoms was pain, primarily of musculo-skeletal origin [2]. Indeed, experience of musculo-skeletal symptoms (including symptoms of pain) became a necessary element in the definition of Fukuda's chronic multisymptom condition used to label the poor health described by veterans of the Gulf War [3]. Pain can therefore be seen as an important factor in military health.

However, pain is not uncommon in either the general population or in non-deployed military cohorts [4]. Pain in military cohorts is primarily associated with injuries arising from increased levels of physical activity experienced in military training [5]. Any investigation into the relationship between deployment to the Gulf War and subsequent experience of pain must account for this. The investigations are also made more difficult through the reliance on a self-reported measurement of health outcome. Studies that have attempted to clinically confirm self-reported pain have shown little association between the self-reported and clinical measurements, possibly due to the sometimes transitory nature of pain [6].

This paper describes a systematic review of studies comparing the prevalence of symptoms of pain in veterans of the Gulf War with its prevalence in a comparison group who were not deployed to the Gulf (non-Gulf veterans).

## Methods

### Searching

The methods employed in the systematic review have been described in another paper [7] and are summarised here. 5387 studies from the period January 1990 to May 2001 were identified for possible inclusion by searching through databases (EMBASE, Medline, ASSIA, SIGLE, PsycINFO, CancerLit, HealthSTAR, Dissertation Abstracts, Current Contents, Health and Psychosocial Instruments, CINAHL and Biological Abstracts) and websites and by contacting researchers in the field. Studies were eligible for inclusion if they contained data on military, medical or peace-keeping personnel who were deployed to the Gulf War together with a comparison group which differed in its level of exposure. Abstracts of 2296 references that remained eligible were examined by two members of the research team. Studies were excluded if they measured simulated exposures, if they measured non-health related outcomes or if the subjects were inhabitants of the Persian Gulf rather than deployed military personnel. Studies that examined pain within groups of Gulf veterans that had experienced differential exposures whilst in the Gulf, e.g. exposure to the smoke from oil-well fires, were also excluded from this review.

All included studies were categorised by health outcome, one of which was pain. Any site of the body where pain was reported was included within the review. The definition of pain in this review therefore included any symptom or condition that included the word 'pain', e.g. 'chest pain', 'joint pain' and 'muscle pain'. It did not include any symptom or condition that is frequently associated with pain, such as arthritis, fibromyalgia or headache. Thirteen papers were identified from this search.

An updated electronic search of the literature from January 2001 to May 2004 was completed which identified a further 538 references. Of databases searched in 2001, CancerLit and HealthStar were now incorporated into Medline whilst Dissertation Abstracts and Health and Psychosocial Instruments were no longer available. Two databases not searched in 2001, the Web of Knowledge Databases and the Science and Social Science Citation Indexes were included in this updated search. Of 538 potentially relevant references, 105 were selected from the abstract (or title if no abstract) as potential research studies with a relevant comparison group. From these, seven papers were identified and therefore this review contains 20 papers that fulfilled our inclusion criteria and which contained data relating to pain both in Gulf War veterans and non-Gulf veterans. These non-Gulf veterans may or may not have been deployed elsewhere on active duty.

### Data extraction

Data relating to the studies' main hypotheses and to methodological quality were extracted independently by two members of the research team onto pre-designed data extraction forms. Information on methodological quality of the individual studies included the response or follow-up rate, the potential of selection bias in the sampling of subjects, the potential bias in the measurement of outcomes, and the availability of data on confounders and the controlling for such variables.

### Statistical analysis

Meta-analysis statistically combines and analyses data from separate studies with the aim of appraising the evidence objectively, providing a more precise estimate of effect and exploring any heterogeneity between the results of individual studies [8]. A summary odds ratio was calculated with a random effects model using the DerSimonian and Laird method [9]. The estimate of heterogeneity between studies was taken from the inverse variance fixed effect model. All analyses were performed using the "metan" command [10] in Stata Version 9 (Stata Corporation, College Station, TX, USA). We chose to use this approach because of our a priori view that the studies were inherently heterogeneous.

## Results

### Studies identified

The twenty studies that fulfilled the inclusion criteria are described in Table 1[3,11-29]. Three further studies were also identified but excluded on the grounds that their inclusion would have lead to duplication of data within the results [30-32]. Gray *et al *[30] presented data relating to the same study population described by Knoke *et al *[24] but included fewer pain outcomes, therefore data reported by Knoke *et al *are utilised. Wolfe *et al *[31] reported overall bodily symptom scores (not specifically pain), therefore data relating to pain outcomes quoted in Proctor *et al *[18] have been included instead. Finally, since Nisenbaum *et al *[32] presented data on the same study population as Fukuda *et al *[3] only data from Fukuda *et al *are included.

### Muscle pain

Prevalence of 'muscle pain' was measured in eight of the studies (Table 2). Veterans were asked to report whether they had "muscle pain" [3,20,23,25,29], "unusual muscle pain" [24,27] or "muscle pain/weakness" [28]. Ishoy *et al *[20] presented no clear numerical data on muscle pain and therefore the results could not be included in the meta-analysis, but instead stated that there were no statistically significant differences in the reporting of pain between the Gulf veterans and non-Gulf veterans. The overall reported prevalence of muscle pain was highest amongst the Gulf War veteran study sample of Kelsall *et al *[29], but the non-Gulf veterans also reported a particularly high prevalence which therefore resulted in a weak association between deployment and symptoms. Conversely, the prevalence of muscle pain reported both by Gulf and non-Gulf veterans was unusually low in the study sample of Simmons *et al *[28]. These authors were the first to measure health outcomes using an open-ended question enquiring about any new medical problems or changes in general health since 1990 instead of relying on respondents to tick relevant boxes for pre-defined categories of symptoms. This method of data collection might minimise over-reporting of symptoms.

The meta-analysis provided a summary estimate of OR 3.06 (95% CI 2.18–4.30) reflecting an independent association between deployment to the Gulf War and subsequent reporting of muscle pain. Significant statistical heterogeneity (χ^2 ^= 173.1 df = 6 P < 0.001) was found between the studies.

### Joint pain

Table 3 lists the twelve studies that included joint pain as a reported outcome. Ishoy *et al *[20] presented no clear numerical data but again stated that there were no significant differences in the reporting of joint pain between Gulf and non-Gulf veterans. Most of the studies reported that approximately 30–40% of Gulf veterans experienced symptoms of joint pain, making it one of the most common sites of pain. A particularly large proportion (74%) of Gulf War veterans reported joint pain in the study sample of Sostek *et al *[12]. The authors themselves suggested that a reporting bias might be present in the study as a greater proportion of Gulf War veterans also reported a change of colour of their fingernails which was included as a control symptom.

Overall the odds of reporting joint pain was nearly three times greater amongst the Gulf veterans as summarised by the meta-analysis (OR 2.81; 95%CI 2.31 – 3.42). There was significant statistical heterogeneity between the study results (χ^2 ^= 144.7 df = 10 P < 0.001).

### Chest or heart pain

Chest or heart pain was reported in seven of the studies (Table 4). Data from Cherry *et al *[26] could not be included in the meta-analysis since they were presented as mean scores rather than prevalence estimates. Data from Proctor *et al *[18] were not included in the meta-analysis because the prevalence of symptoms in the non-Gulf veterans was zero and did not allow for a meaningful comparison. An unusually low prevalence of symptoms was again observed in the study sample of Simmons *et al *[28], although the ratio measure arising from the data was consistent with those across the studies. The meta-analysis provided an overall summary estimate of OR 2.52 (95%CI 2.23–2.85). The test for heterogeneity was not significant (χ^2 ^= 7.4 df = 4 p= 0.115) which suggests that the estimates from individual studies were consistent with each other.

### Back pain

Table 5 shows the prevalence estimates reported by six studies investigating back pain as an outcome. It was not possible to include data from the Goss Gilroy study [17] in the meta-analysis since no indication of exact sample size was provided. The overall summary estimate was OR 1.58 (95%CI 1.23–2.04). Again, there was significant statistical heterogeneity between the study results (χ^2 ^= 49.8 df = 3 P < 0.001).

### Abdominal pain

Table 6 summarises the results of six studies that investigated the association between Gulf deployment and abdominal pain. The mean symptom scores reported by Cherry *et al *[26] could not be included in the meta-analysis. The reported prevalence of this symptom in Gulf War veterans varied greatly between studies, ranging from three percent to seventy percent. This particularly high prevalence reported by Sostek *et al *[12] will certainly have contributed to the statistical heterogeneity between study estimates, even though the study carried least weight in the meta-analysis. The overall summary estimate generated by the meta-analysis (OR 3.23; 95%CI 2.31–4.51) was therefore not surprisingly associated with significant statistical heterogeneity between the studies (χ^2 ^= 29.8 df = 4 P < 0.001).

### Other sites of pain

Four studies reported the prevalence of symptoms of either body pain, widespread pain or general aches whilst three studies measured scores of bodily pain using the SF-36[11,13,15,16,19,25,26]. A meta-analysis was considered inappropriate to summarise the results due to the variation in measurement of the symptom. All seven studies reported a positive association between Gulf deployment and painful symptoms, however those symptoms were measured. For example, 19.2% of Gulf veterans versus 9.6% of non-Gulf veterans reported chronic widespread pain in the sample of Peloso *et al *[16]. Similarly 12.2% of Gulf veterans versus 6.5% of non-Gulf veterans reported widespread pain in the sample of Cherry *et al *[26].

## Discussion

Nineteen of the twenty primary studies identified by this review recorded that a greater proportion of veterans of the Persian Gulf War of 1991 reported painful symptoms compared to other military service personnel who were not deployed to the Gulf War. For all five sites of bodily pain, each of the summary estimates from the meta-analyses indicated deployment to the Gulf War was associated with increased odds of reporting painful symptoms. Gulf deployment was most strongly associated with abdominal pain, with Gulf veterans being more than three times more likely to report such pain.

Unfortunately the majority of studies included in this review did not investigate whether Gulf War veterans report more symptoms of severe pain than non-Gulf veterans. Only Kang *et al *and Kelsall *et al *reported prevalence estimates separately for moderate to severe symptoms. Kang *et al *found that a greater proportion of the Gulf veterans than non-Gulf veterans reported more severe symptoms of joint pain, but they did not differ from non-Gulf veterans in the severity of symptoms of back, muscle or abdominal pain [23]. Kelsall *et al *reported that more of the Gulf veterans suffered from general muscle aches and pains that were more severe in nature but that their degree of symptoms of back or joint pain was the same as non-Gulf veterans [29].

Statistical heterogeneity between study estimates for a particular site of pain was significant (with the exception of chest pain). Variation in each of the following characteristics across studies probably contributed to this heterogeneity: sampling strategy (single military units versus stratified random samples), degree of differential response rates between Gulf and non-Gulf veterans, method of symptom ascertainment, measured period of prevalence and specific definition of symptoms.

At least the statistical heterogeneity that arose probably reflects heterogeneity in the strength of association rather than the direction of association.

The prevalence of painful symptoms amongst Gulf War veterans was most often reported to be between approximately 20% and 40% depending on the site of pain and exact definition of measurement. In contrast, a recent population-based survey of young adults aged 18 to 25 years in the UK observed that 66.9% (95%CI 63.7% to 70.1%) reported any pain within the previous six months, although a low response rate (37%) means the estimates should be interpreted with caution [33]. The prevalence of pain amongst military personnel when compared to the general population might be expected to be relatively low due to a "healthy worker" effect, but conversely the increased risk of pain received through injuries during military training may contribute to the prevalence of pain in military populations. This highlights the need in study samples for a relevant comparison between veterans deployed to the Gulf War and either veterans deployed elsewhere or non-deployed military personnel.

### Limitations of primary research

#### Sampling of participants

In a cross-sectional survey it is important to derive a random sample of all those subjects who are potentially eligible in order to generate a representative sample of the larger population of interest. Those studies which selected a random sample of veterans from either US, British, Canadian, Danish or Australian military personnel databases are likely to have fulfilled this criterion [17,20,21,23,26,28,29]. However, those studies which sampled more opportunistically from individual military units are more prone to selection bias [3,11,12,18].

#### Response bias

In general, most of the studies achieved a satisfactory response rate amongst veterans of the Gulf War. However the response rate amongst non-Gulf veterans unfortunately tended to be systematically lower in most studies for which data were available. Differences in response rates between the exposed and unexposed groups can lead to bias if the responders are systematically different to non-responders. Unwin *et al *[21] intensively followed up a random selection of non-responders and found that those with more symptoms responded earlier but there was no significant interaction between deployment, late response and health outcome. So the prevalence estimate of symptoms might be a biased overestimate, but relative measures of effect as reported in this review should be less prone to bias. Kelsall *et al *[29] also suggested that response bias is unlikely to fully explain any differences observed between Gulf and non-Gulf veterans. They reported that odds ratios from a prediction model which assumed full participation and accounted for age, rank and service were only marginally lower than corresponding odds ratios observed for participants.

#### Symptom measurement

All of the studies relied on the veterans' self reported symptoms of pain which would be prone both to random measurement error and more importantly to measurement bias. Two studies included symptom items in their questionnaires which were not thought to have any physiological basis but were designed to estimate the level of over-reporting of symptoms amongst Gulf War veterans. For example Knoke *et al *[24] found that 1.2% of Gulf veterans versus 0.2% of non-Gulf veterans reported symptoms of 'earlobe pain', whilst Sostek *et al *[12] reported a significantly greater proportion of Gulf veterans reported a 'change in the colour of fingernails'. These results suggest that at least some of the association between Gulf deployment and reporting of painful symptoms might be explained by systematic over-reporting of symptoms amongst Gulf veterans.

In an attempt to minimise the measurement error and possible bias that might be associated with the reliance on symptom checklists, Simmons *et al *[28] introduced the use of open-ended questions enquiring about any new medical problems or changes in general health since 1990. This method of data collection was indeed associated with lower overall prevalence of symptoms but still demonstrated greater reporting of symptoms amongst Gulf War veterans relative to non-Gulf veterans.

#### Confounding

A few of the earliest studies did not attempt to control for potential confounders in any way and therefore may have inflated estimates of risk [3,11,12]. Some studies accounted for the effect of gender by restricting their analysis to a single sex [21,22,24,28,29], whilst some studies made adjustments for a number of confounding variables in the analysis of the data [18,19,21,22,25,27-29]. However, the later and larger studies tended to control for potential confounders more thoroughly in the sampling design of the study by matching veterans on age, sex and at least some aspect of military status [13-17,20-22,26,28,29].

Since the meta-analyses are based on the raw prevalence data from each study, potential confounders could only be partially accounted for in the resulting summary odds ratios if individual studies stratified both the Gulf and non-Gulf samples on age, sex or military status. To estimate the size of the possible effect of confounding on our reported summary estimates, it would be useful to compare the unadjusted and adjusted results from any of the primary studies. However very few of the primary studies report both raw and adjusted results. Unwin *et al *reported an unadjusted OR of 2.8 (95%CI 2.5–3.2) for joint pain in male Gulf veterans versus era controls which was reduced to an OR of 2.2 (95%CI 2.0–2.6) after adjusting for age, smoking, alcohol consumption, marital status, educational attainment, rank, employment status and civilian or military status on follow-up. It might seem reasonable to assume that our unadjusted summary ORs arising from the meta-analyses might be similarly overestimating the true association between painful symptoms and Gulf deployment.

### Strengths and limitations of this review

This review benefits from a sensitive search strategy based on both published material and on grey literature such as conference abstracts and preliminary reports. Furthermore, inclusion and exclusion criteria were independently assessed by two reviewers. However, failure to identify some studies is always a possibility in systematic reviews. The majority of the primary studies that we identified reported Gulf deployment to be independently associated with symptoms of pain. The absence of many studies with negative findings raises the possibility of the existence of publication bias. However in order to affect the weighted summary estimates derived from the meta-analyses, any statistically significant negative results that are currently missing from the review would have to have been based in large study samples and these would have been more likely to be published. Therefore the likelihood of publication bias being present which would actually alter the conclusions of the review is small.

This review could only investigate symptoms of pain in sites reported by the primary studies. Published papers might have been limited to reporting only the most frequently recorded symptoms rather than all measured symptoms [14,21,22,29], and therefore the association between Gulf deployment and symptoms of pain in other unreported sites is unknown. However, given the consistency in the results for all measured sites of pain included in this review, it might seem unlikely that Gulf deployment would have a dramatically different association with any unreported site of pain.

This review has been limited to investigating the association between Gulf deployment versus non-deployment and reporting of pain. We chose not to examine the association between specific environmental exposures of the war (e.g. threat of chemical warfare agents, non-routine immunisations) and reporting of symptoms due to the problems associated with the inaccuracy of such self-reported exposures.

In this review we were not attempting to measure the possible underlying biological or socio-cultural mechanisms which could explain the observed association between Gulf deployment and symptoms of pain. However, the experience of being deployed into a potentially life threatening situation is obviously extremely stressful, and psychological stress can manifest itself in a range of physiological symptoms, including pain [34].

## Conclusion

The results of this systematic review support the hypothesis that a higher proportion of veterans of the Persian Gulf War of 1991 have reported symptoms of pain than comparison groups of military personnel. Gulf deployment was most strongly associated with abdominal pain, with Gulf veterans being more than three times more likely to report such pain. However, the methodological quality of the primary studies varied greatly and the summary estimates from meta-analyses were often associated with statically significant heterogeneity. At least some of the observed association might be explained by response bias, measurement bias and confounding. Even if the point estimates of relative risk observed in this review are somewhat inflated due to these limitations of the data, it is still clear that Gulf War veterans continue to suffer from poorly understood painful symptoms many years after returning from the conflict. These findings are consistent with the generally increased reporting of all symptoms (for example multiple chemical sensitivity, chronic fatigue, post traumatic stress disorder, common mental disorder) by veterans of the Gulf War of 1991 when compared to other military groups [7,35].

## Competing interests

The author(s) declare that they have no competing interests.

## Authors' contributions

GL, FD and HT conceived the study. All authors participated in the design of the study. AW performed the literature search. NS, HT, FD and GL extracted the data. NS initially drafted the manuscript and performed statistical analyses. HT repeated the analyses with updated data and prepared the manuscript for publication. All authors read and approved the final manuscript.

## Pre-publication history

The pre-publication history for this paper can be accessed here:



## Figures and Tables

**Table 1:** Characteristics of studies that have investigated the association between deployment to the Gulf War and symptoms of pain amongst veterans

**Study**	**Sample**	**Study design**	**Measured outcomes**	**Response rate and bias**	**Confounding**	**Notes**
Sutker 1993	215 GWV 44 NGV Sampled from 5 National Guard and Army Reserve units as part of debriefing programme	Questionnaire administered by VA staff 4–10 months after return from Operation Desert Shield/Storm	General aches and pains as measured by the Health symptom checklists	70% GWV response (306 eligible) 91 GWV excluded from analysis for failure to complete the majority of measures administered, however, they 'did not differ on descriptive variables' from those that were analysed. Number of eligible NGVs unclear	Bonferonni adjustment showed no sig. effects for sex/race although women and non-white participants tended to report more health complaints	Symptom presence included the report of the symptom at least once a week over the past 30 days
Sostek 1996	57 GWV 44 NGV members of a single National Guard Unit	Questionnaire survey	Joint pain Abdominal pain (infra or supra umbilical location)	62% GWV (92 sampled) 100% NGV (44 sampled) Unclear what percentage of the unit was sampled	No adjustments were made for confounding. Groups were said to be similar on a range of socio-demographic variables with the exception of age. Gulf veterans were significantly older.	Authors suggest that bias may be present in their study as a significantly higher proportion (p≤0.05) of Gulf veterans report experiencing a 'change in the colour of fingernails' (a control symptom)
Iowa 1997	1896 GWV 1799 NGV DoD Manpower Data Center used to create a stratified random sample from 28,968 military personnel from Iowa	Cross-sectional telephone interview survey Sept 1995 – May 1996	Bodily pain	78% GWV (2421 eligible) 73% NGV (2465 eligible) Those who were regulars, enlisted, navy or coast guard, aged up to 25 years and black or other ethnic background less likely to participate	Controlled for stratification variables: military service (regular/National Guard), age, sex, race, branch of service and rank	Response reliability for 165 interviews kappa 0.39–0.79
Doebbeling 2000	see Iowa 1997	see Iowa 1997	Pain or aches in more than 1 joint Back pain	see Iowa 1997	see Iowa 1997	
Voelker 2002	See Iowa 1997	see IOWA 1997	Bodily pain on SF-36	see Iowa 1997	See Iowa 1997	Effect size is the standardised mean difference on the bodily pain subscale of the SF36
Peloso 2002	See Iowa 1997	See Iowa 1997	Chronic widespread pain	See Iowa 1997	See Iowa 1997	Outcome – pain present in last year and last month, moderate or greater severity and involvement of upper and lower extremities and trunk
Fukuda 1998	1163 GWV 2538 NGV Sampled veterans were active duty members of the Air Force, from Florida or Pennsylvania and who were on base at the time of the survey	Cross-sectional population survey Jan – March 1995	Joint pain Muscle pain Chest pain	Veterans were not sampled according to Gulf status so no differential response rates are given. Unit response rate 35% – 73%	No report of adjustment made for potential confounders.	
Goss Gilroy 1998	3113 Canadian GWV 3439 Canadian NGV Sampled from Department of National Defence human resources data files	Cross-sectional survey	Serious trouble with back pain	73% GWV (4262 eligible) 60% NGV (5699 eligible)	NGVs matched on sex, age group and regular/reserve status	Only data for male veterans is presented.
Proctor 1998	186 GWV from Fort Devens 66 GWV from New Orleans 48 Germany deployed veterans	Cohort study Spring 1994 – Autumn 1996	Joint pain Chest pain	53% Fort Devens (353 sampled, 2949 eligible) 34% New Orleans (194 sampled, 928 eligible) 51% Germany deployed. Participants were recruited after taking part in a previous study	Published odds ratios were weighted for sampling design and adjusted for age, sex and education	
Proctor 2001	See Proctor 1998	See Proctor 1998	Chronic back pain Bodily pain on SF-36	See Proctor 1998	See Proctor 1998	
Ishoy 1999	686 GWV 231 NGV Selected from Danish Armed Forces personnel database	Cross-sectional survey Feb 1997-Jan 1998	Joint pain Muscle pain	84% GWV (821 eligible) 58% NDV (400 sampled) Non-response meant GWV more likely to be older males	Controls matched for gender, age and profession	
Unwin 1999	3284 GWV 1815 Bosnia veterans 2408 Era controls Random sample taken from the UK MoD database Service men only	Cross-sectional postal survey August 1997-Nov 1998	Pain without redness or swelling in several joints Chest pain	70% GWV (4250 eligible) 62% Bosnia (4250 eligible) 63% Era (4246 eligible) Responders were significantly older and more likely to be in service but didn't differ on SF36 ratings	Sample was stratified by service, status, sex, age, rank and fitness. Analyses restricted to male veterans. Potential confounders (age, marital status, rank, education, employment, still serving or discharged, smoking, alcohol consumption and GHQ score) are adjusted for by logistic regression	
Unwin 2002	236 GWV 217 Bosnia veterans 192 Era controls Service women only	See Unwin 1999	Pain without redness or swelling in several joints	72% GWV 66% Bosnia 58% Era	Analyses restricted to female veterans.	
Kang 2000	11441 GWV 9476 NGV Stratified random sample taken from the DoD Manpower Data Center	Population based survey 1995–1997	Joint pain Muscle pain Back pain Abdominal pain	76% GWV (15000 eligible) 63% NGV (15000 eligible) Non responders were more likely to be younger, unmarried, non-white and enlisted	Estimates were weighted to control for stratification variables: gender and unit component	
Knoke 2000	524 GWV 935 NGV Sampled from active duty US Naval mobile construction battalion personnel (Seabees). Personnel had to be in residence at either Port Hueneme or Gulfport	Questionnaire survey carried out in 1994	Pains in lower back Pains in heart/chest Moving joint pain Unusual muscle pain Chest pain Abdominal pain Earlobe pain	65% GWV 46% NGV	Analyses restricted to male veterans.	'Earlobe pain' was included as a validity symptom as it was thought not to have a physiologic basis
Steele 2000	1435 GWV 409 NGV DoD Manpower Data Center used to create a stratified random sample from 16,566 military personnel from Kansas	Population based telephone survey Feb – Aug 1998	Joint pain Muscle pain Body pain (hurts all over) Abdominal pain or cramping Moderate/multiple pain symptoms	65% response (3138 eligible) Overall, Gulf and female veterans were more likely to respond.	Published odds ratios are adjusted for sex, age, income level and education level.	Women and reservists deliberately over sampled due to increased reporting of ill health in those groups. Veterans had to be either separated or retired from military or currently serving in the reserves.
Cherry 2001	8210 GWV 3981 NGV Random sample taken from the UK MoD database	Cross-sectional survey Dec 1997-Sept 1999	Stomach pain Pain in your chest Widespread pain	86% GWV (9505 eligible) 84% NGV (4749 eligible) Non responders were younger	NGV sample stratified by sex, age, service and rank to frequency match GWV sample.	Widespread pain defined as "axial skeletal and contra lateral body pain" for at least 24 hours in the past month
Gray 2002	3831 GWV 4933 deployed elsewhere 3104 non-deployed All active duty Seabees regardless of whether still in service at time of study identified through Defense Manpower Data Center	Cross-sectional survey May 1997 – May 1999	Unusual muscle pain Joint pain Chest pain Stomach pain/ulcer	Total eligible 18,945 64% response Respondents more likely to be reservists, married, Caucasian, and deployed elsewhere.	Published odds ratios adjusted for age, gender, active-duty/reserve status, ethnicity, smoking, alcohol use.	Reliability for self-reported physician-diagnosed medical conditions in 519 subjects retested 6 months apart: Kappa = 0.6
Simmons 2004	23,358 GWV 17,730 NGV Stratified random sample from UK MoD database	Cross-sectional postal survey Aug 1998-March 2001	Muscular pain/weakness Chest pains/tightness	48% response overall. Pain data available for: 45% GWV (51581 eligible) 34% NGV (51688 eligible)	Analysis restricted to male veterans. Published odds ratios adjusted for age, service, rank, service status at time of survey, alcohol and smoking	
Kelsall 2004	1456 GWV 1588 NGV Stratified random sample from Australian Defence Force database	Cross-sectional postal survey Aug 2000-April 2002	General muscle pain Pain without redness or swelling in several joints Low back pain	78% GWV (1871 eligible) 54% NGV (2924 eligible) Non responders were younger and of lower rank.	Analyses restricted to male veterans. Published odds ratios adjusted for sex, 3 yr age band, service and rank.	

GWV – Gulf War veteran

NGV – Non-Gulf veteran

DoD – US Department of Defense

MoD – British Ministry of Defence

**Table 2:** The association between deployment to the Gulf War and muscle pain amongst veterans

**Study**	**Period of prevalence estimate**	**Prevalence GWV**	**Prevalence NGV**	**OR (95% CI)**	**% weight in meta-analysis**
Fukuda 1998	Current symptoms Symptoms for 6 months	20% 18%	8% 6%	2.88 (2.35–3.53)	15.0
Ishoy 1999	No numerical data				
Kang 2000	Time period unclear	33%	17%	2.41 (2.25–2.57)	15.8
Knoke 2000	Current symptoms	7.1%	1.8%	4.10 (2.29–7.36)	10.8
Steele 2000	Symptoms past 12 months	21%	6%	4.08 (2.67–6.23)	12.8
Gray 2002	Symptoms past 12 months	22.6%	DEV 6.9% NDV 5.7%	3.96 (3.47–4.53)	15.5
Simmons 2004	Symptoms since 1990	2.0%	0.4%	5.07 (3.95–6.52)	14.6
Kelsall 2004	Symptoms past month	52%	46%	1.27 (1.10–1.47)	15.5
**Summary OR (95% CI)**				**3.06 (2.18–4.30)** **Heterogeneity χ^**2 **^= 173.1, df = 6, P < 0.001**	

GWV – Gulf War veterans; NGV – non-Gulf veterans.

OR – odds ratio. Individual study ORs based on published prevalence data and not adjusted for confounders.

Summary OR – weighted average of all individual study ORs, derived from "metan" command in Stata version 9.0 using a random effect model and DerSimonian and Laird method.

Fukuda 1998: data on current symptoms used in meta-analysis.

Gray 2002: DEV – deployed elsewhere veterans, NDV – non-deployed veterans; GWV vs DEV compared in meta-analysis.

**Table 3:** The association between deployment to the Gulf War and joint pain amongst veterans

**Study**	**Period of prevalence estimate**	**Prevalence GWV**	**Prevalence NGV**	**OR (95% CI)**	**% weight in meta-analysis**
Sostek 1996	Current symptoms	74%	30%	6.68 (2.78–16.1)	3.5
Doebbeling 2000	Symptoms past 12 months	37%	16%	3.08 (2.64–3.61)	11.2
Fukuda 1998	Current symptoms Symptoms for 6 months	36% 18%	13% 6%	3.77 (3.19–4.45)	11.1
Proctor 1998	Symptoms past month	FD 33% NO 32%	16%	2.41 (1.08–5.39)	4.0
Ishoy 1999	No numerical data				
Unwin 1999	Symptoms past month	32.2%	Bosnia 13.8% Era 14.4%	2.82 (2.46–3.23)	11.4
Unwin 2002	Symptoms past month	27.8%	Bosnia 12.7% Era 10.8%	3.16 (1.85–5.40)	6.4
Kang 2000	Time period unclear	45%	27%	2.21 (2.09–2.34)	11.9
Knoke 2000	Current symptoms	14.1%	4.7%	3.33 (2.25–4.92)	8.2
Steele 2000	Symptoms past 12 months	37%	15%	3.35 (2.50–4.49)	9.5
Gray 2002	Symptoms past 12 months	37.9%	DEV 16.8% NDV 13.6%	3.02 (2.74–3.33)	11.6
Kelsall 2004	Symptoms past month	35%	29%	1.30 (1.12–1.52)	11.2
**Summary OR (95% CI)**				**2.81 (2.31–3.42)** **Heterogeneity χ^**2 **^= 144.7, df = 10, P < 0.001**	

GWV – Gulf War veterans; NGV – non-Gulf veterans.

OR – odds ratio. Individual study ORs based on published prevalence data and not adjusted for confounders.

Summary OR – weighted average of all individual study ORs, derived from "metan" command in Stata version 9.0 using a random effect model and DerSimonian and Laird method.

Fukuda 1998: data on current symptoms used in meta-analysis.

Proctor 1998: FD – Fort Devens sample, NO – New Orleans sample; combined prevalence in FD and NO vs NGV compared in meta-analysis

Unwin 1999/2002: GWV vs Era controls compared in meta-analysis.

Gray 2002: DEV – deployed elsewhere veterans, NDV – non-deployed veterans; GWV vs DEV compared in meta-analysis.

**Table 4:** The association between deployment to the Gulf War and chest pain amongst veterans

**Study**	**Period of prevalence estimate**	**Prevalence GWV**	**Prevalence NGV**	**OR (95% CI)**	**% weight in meta-analysis**
Fukuda 1998	Current symptoms Symptoms for 6 months	15% 13%	7% 5%	2.33 (1.87–2.91)	18.3
Proctor 1998	Symptoms past month	FD 6% NO 3%	0%		
Unwin 1999	Symptoms past month	25.3%	Bosnia 13.2% Era 11.8%	2.53 (2.19–2.93)	27.7
Knoke 2000	Current symptoms	20.2%	10.6%	2.79 (1.61–4.84)	4.5
Cherry 2001	Symptoms past month	Mean score approx 3.7	Mean score approx 2.0		
Gray 2002	Symptoms past year	16.0%	DEV 6.1% NDV 5.1%	2.93 (2.54–3.39)	28.0
Simmons 2004	Symptoms since 1990	1.7%	0.8%	2.14 (1.77–2.60)	21.5
**Summary OR (95% CI)**				**2.52 (2.23–2.85)** **Heterogeneity χ^**2 **^= 7.4, df = 4, P = 0.115**	

GWV – Gulf War veterans; NGV – non-Gulf veterans.

OR – odds ratio. Individual study ORs based on published prevalence data and not adjusted for confounders.

Summary OR – weighted average of all individual study ORs, derived from "metan" command in Stata version 9.0 using a random effect model and DerSimonian and Laird method.

Fukuda 1998: data on current symptoms used in meta-analysis.

Proctor 1998: FD – Fort Devens sample, NO – New Orleans sample; data not included in meta-analysis because prevalence in reference group is zero.

Unwin 1999: GWV vs Era controls compared in meta-analysis.

Gray 2002: DEV – deployed elsewhere veterans, NDV – non-deployed veterans; GWV vs DEV compared in meta-analysis.

**Table 5:** The association between deployment to the Gulf War and back pain amongst veterans.

**Study**	**Period of prevalence estimate**	**Prevalence GWV**	**Prevalence NGV**	**OR (95% CI)**	**% weight in meta-analysis**
Doebbeling 2000	Symptoms past 12 months	37%	16%	2.25 (1.92–2.64)	23.0
Goss Gilroy 1998	Time period unclear	Age 20–44 yrs 20.2% Age 45–64 yrs 25.6%	Age 20–44 yrs 15.3% Age 45–64 yrs 19.9%		
Proctor 2001	Symptoms past month	21.4%	21.7%	0.97 (0.43–2.18)	7.0
Kang 2000	Time period unclear	44%	30%	1.83 (1.73–1.94)	25.0
Knoke 2000	Current symptoms	50.9%	40.4%	1.53 (1.23–1.90)	21.4
Kelsall 2004	Symptoms past month	52%	49%	1.14 (0.99–1.32)	23.5
**Summary OR (95% CI)**				**1.58 (1.23–2.04)** **Heterogeneity χ^**2 **^= 49.8, df = 4, P < 0.001**	

GWV – Gulf War veterans; NGV – non-Gulf veterans.

OR – odds ratio. Individual study ORs based on published prevalence data and not adjusted for confounders.

Summary OR – weighted average of all individual study ORs, derived from "metan" command in Stata version 9.0 using a random effect model and DerSimonian and Laird method.

**Table 6:** The association between deployment to the Gulf War and abdominal pain amongst veterans

**Study**	**Period of prevalence estimate**	**Prevalence GWV**	**Prevalence NGV**	**OR (95% CI)**	**% weight in meta-analysis**
Sostek 1996	Current symptoms	70%	9%	23.5 (7.27–76.1)	6.5
Kang 2000	Time period unclear	23%	12%	2.19 (2.03–2.36)	32.2
Knoke 2000	Current symptoms	3.4%	1.3%	2.74 (1.31–5.73)	12.6
Steele 2000	Symptoms past 12 months	15%	4%	4.33 (2.57–7.28)	18.2
Cherry 2001	Symptoms past month	Mean score approx 4.0	Mean score approx 2.5		
Gray 2002	Symptoms past 12 months	13.4%	DEV 5.1% NDV 4.4%	2.87 (2.45–3.36)	30.5
**Summary OR (95% CI)**				**3.23 (2.31–4.51)** **Heterogeneity χ^**2 **^= 29.8, df = 4, P < 0.001**	

GWV – Gulf War veterans; NGV – non-Gulf veterans.

OR – odds ratio. Individual study ORs based on published prevalence data and not adjusted for confounders.

Summary OR – weighted average of all individual study ORs, derived from "metan" command in Stata version 9.0 using a random effect model and DerSimonian and Laird method.

Gray 2002: DEV – deployed elsewhere veterans, NDV – non-deployed veterans; GWV vs DEV compared in meta-analysis.
